# Supplementary material for: Aging aggravates aortic aneurysm and dissection via miR-1204-MYLK signaling axis in mice
Source: Nat Commun. 2024 Jul 16;15:5985. doi: 10.1038/s41467-024-50036-2 (PMC11252124; doi:10.1038/s41467-024-50036-2)
Supplement: Supplementary file 1 — Supplementary Information [file 41467_2024_50036_MOESM1_ESM.pdf]

Supplementary information for

Aging aggravates aortic aneurysm and dissection via miR-1204—MYLK signaling axis

This file includes:

Supplemental Table 1 to Supplemental Table 6

Supplemental Figure 1 to Supplemental Figure 9

Supplemental Tables

Supplemental Table 1 Clinical characteristics (young versus old)

**Characteristics**	**Young normal （70）**	**Older normal （88）**	***P***	**Young patient （116）**	**Older Patient （156）**	***P***
Age, y	42.1±5.6	62.1±4.5	<0.0001	40.3±6.6	64.2±8.6	<0.0001
Male,n(%)	52(74.2)	64(72.7)	0.83	98(84.5)	122(78.2)	0.19
Smoking,n(%)	19(27.1)	38(43.1)	0.037	42(36.2)	71(45.5)	0.12
Hypertension,n(%)	-	-	-	75(64.7)	121(77.6)	0.019
Diabetes Mellitus,n(%)	-	-	-	2(1.7)	15(9.6)	0.0095
CTD,n(%)	-	-	-	8(6.9)	2(1.3)	0.021
TG, mmol/L	1.11±0.48	1.3±0.75	0.34	1.25±0.7	1.28±0.66	0.91
TC, mmol/L	3.72±0.81	3.7±0.79	0.93	3.73±0.52	3.86±0.44	0.58
LDL, mmol/L	2.38±0.69	2.36±0.74	0.94	2.41±0.42	2.29±0.32	0.51
HDL, mmol/L	1.14±0.22	1.08±0.25	0.46	1.11±0.22	1.03±0.16	0.36
Uric Acid, umol/L	389.9±156.7	392.2±153.3	0.96	359±132.2	346.1±134.6	0.59

Data are presented as means ± standard deviation or the number of patients (n, %). CTD, Connective Tissue Diseases; TG, triglyceride; TC, total cholesterol; LDL, low-density lipoprotein; HDL, high-density lipoprotein. The unpaired two-tailed Student’s t-test or the Mann–Whitney *U* test was applied to evaluate statistical significance for continuous variables

with or without normal distribution, respectively. The Chi-square test was used to evaluate the statistical significance between the proportions of the two groups.

Supplemental Table 2 Mice body weight in AngII-induced AAD model

	Before miRNA injection(g)	After miRNA injection(g)	After AngII administration(g)
miR-control	24.3±0.9	27.7±0.9	33.1±1.1
miR-1204	25.1±1.2	28.1±0.8	32.6±0.9
miR-control+AngII	25.0±0.9	27.5±1.3	32.5±0.8
miR-1204+AngII	25.3±0.3	27.9±1.1	33.5±1.2

Data are presented as means ± standard deviation. AngII, Angiotensin II. n=17 for miR-1204+AngII group. n=19 for other groups.

Supplemental Table 3 Mice body weight in BAPN-induced AAD model

	Before BAPN drinking(g)	1 week after BAPN drinking(g)	2 weeks after BAPN drinking(g)	3 weeks after BAPN drinking(g)	4 weeks after BAPN drinking(g)
BAPN+ LNA scr-miR	17.2±1.5	18.1±3.1	21.4±1.8	23.2±1.6	23.3±1.9
BAPN+LNA anti-miR-1204	17.7±1.3	19.5±1.5	21.8±1.6	23.4±1.7	24±1.6

Data are presented as means ± standard deviation. BAPN, β-aminopropionitrile monofumarate. n=15 for BAPN+LNA scr-miR group. n=20 for BAPN+LNA anti-miR-1204 group.

Supplemental Table 4 Primers sequences for qRT-PCR of 112 miR-1204's candidate targets

Primer	Forward	Reserve
GAPDH	GAACGGGAAGCTCACTGG	GCCTGCTTCACCACCTTCT
PLG	CAGCTGGGAGCAGGAAGTAT	CTGTGATATTGGAATGCCCTGC
PPARA	AGATTTCGCAATCCATCGGC	TCAATGCTCCACTGGGAGAC
PRKAR1B	GAAGGGCTGTGAGCTGTACG	CATCATGGGAGTCCGACTGT
PRKCA	AAGAACGTGCACGAGGTGAA	CAGTGTCGGGTCCCTTATCC
PTPMT1	GGACTGGTACCACCGCATC	CAGCTGCTCGACTCCTAGTCT
RAMP2	GTCCTGAATCCCCACGAGGC	GGCAATCTCGCAGGGTGCTA
RAPGEF3	CTGGACACCACTCACCAACA	CCGGTCTCGGATGAGGTTTG
RHOJ	CTGATGAGCTACGCCAACGA	ACATAAGGCACGTGAGGCAT
RLN2	TATGCGGCCGCGAATTAGT	ACTTCAGCTCCTGTGGCAAA
SCARB1	GCTCCCAAAACCCTGTTTGC	GGTCAGCGTTGAGGAAGTGA
ITGB3	CCAACATCTGTACCACGCGA	CCTCTAGTACTCGGGCCTCA
MMP14	GGCTACAGCAATATGGCTACCT	TCATGGTGTCTGCATCAGCTT
PDGFB	TCCTGTCTCTCTGCTGCTACCT	TCCTGTCTCTCTGCTGCTACCT
PDGFRB	TGCTGTTGCTGTCTCTCCTG	TGAGGTTGGTCAGTGTGAGC
ACVR2B	GGAAGGCCCAGCTCATGAAT	TCTCGGCAGCAATGAACTGT
ADAM8	TGGAGCTGTATGTGGTCGTG	GGACCACACGGAAGTTGAGT
ADCYAP1	ATCATGCACAGCAGCGTCTA	CCTCGTTAAGGATCCCGTGG

ADORA1	GCCACCTTCTGCTTCATCGT	GAGAGGGATCTTGACCCGGA
ADRA2B	TACCATCTTCGGCAACGCTC	CCGGAAGTACCAGTAGCCCA
AHSG	AGACAACCGAACTGCGATGA	TGCTGAGGCCACACCTTTAC
AKAP13	CCCCATCTGGGGATGTGAAT	GGCCAGCATCATGCAAACTC
AKT1	GCACAAACGAGGGGAGTACA	AAGGTGCGTTCGATGACAGT
AMOT	GCCCAGCAGATGGTTGAGAT	GACGCGCTGGATTTCTGTCT
ANK2	GCAGCTCAGAAAAGCGACAG	GCAGAATCCACAGAGGACCC
AP3B1	TGAAGCGGATTGTTGGGATGA	AGAGCTCGCTGAAAAGTGCT
PKNOX1	AACCCGATGCAGAAGGAGTG	AATGGCCTGCTTGTCCACAT
SGPL1	GTATGAGCCCTGGCAGCTAA	AATGGGCATCTTCCTGGTGAG
SH3PXD2B	ATTTACCGGCGCTACAGCAA	TATCAGGCGTTTGACAGCCA
SHB	ACTCAGATCCCTTTGATGCC	CAGGTTCGTAAGGGGTGTCA
SIX1	CAATCCCTACCCATCGCCG	TCGGTGTTCTCCCTTTCCTTG
SMTN	TTACAGCTGAGGTTCCAGGC	GCTCTCTTGGTGTCAGAGGG
SMYD1	GGTGAACCATGACTGTTGGC	ACATGGATTTCACTGCCTCA
SRC	AGCCCAAGCTGTTCGGAG	TGTTGACAATCTGGAGCCGC
TAL1	GTTCTTTGGGGAGCCGGATG	TGAAGATACGCCGCACAACT
TAZ	TTCCTGCGTTTCAAGTGGGG	AGTAGGGCGGACTGTTAGGA
TBX20	TTTTGCCAAAGGATTCCGGG	GGGTGAGCGTGCATAGGAAT
TCF21	AGCTCCAACTGCGAGAATGG	CCTCAAGTGGGCGATGTAGC
TFDP2	CCGGCTCTGAACTCTACCAT	ACGACATTCCCATCCGCTTT
UBP1	TTTACTCCACGGAAGCACGG	AGGCTCAAGCCTCATCTCTG

WNT2	AATTTGCCCGCGCATTTGTG	CCTGAGAGTACATGAGCCGC
WNT5A	CGCCCAGGTTGTAATTGAAGC	TGTGGTCCTGATACAAGTGGC
ZDHHC16	GTCTACCTCTGGTTCCTGTGC	TCCAAGCAGCCGTAGTTGTA
ZMIZ1	TGAGCTCCATGAAACCCACT	TTACTCCCCAAACCGTGGTG
POTEE	TGCTACAGGGAGAGCGGC	ACCACTTGCCCATCTTGCTC
ADAMTS15	TCTATGTGCTGGCACCCAAG	GCCACCACGAAATTGTAGCC
CLIC4	TGATTACACCTGGCCATCCC	CATGTTGCTGGGTCGTCCTT
COL18A1	AAAGCAGCCACGAGGTGCAA	CGGTTTCGTCGCAGTCCTGA
COL1A1	ATCACCTGCGTACAGAACGG	AGTAGCACCATCATTTCCACGA
CRELD1	GGTGGGCTCCAAGTGTCTCG	GTAGCCCTCGGCACAGATGC
CXCR2	TGCTGAGCCTGCTGGGAAAC	TGGGCAAGGTCAGGGCAAAG
CYB5R3	CCCAGCTCAGCACGTTGG	CAGCGGGTACTTGATGTCCG
DDR1	CGAGCAGGTCATCGAGAACG	GAACCGATGCAGCTGGGAAA
DHCR7	ATCTGCCATGACCACTTCGG	AGACCCTGCAGCGTGTAAAG
DMD	CCCAGCTCAGCACGTTGG	CAGCGGGTACTTGATGTCCG
DUSP6	CGGAAATGGCGATCAGCAAG	TGTGCGACGACTCGTATAGC
EDN2	CTGGGTGAACACTCCTGAACA	GCCAGTCTGGAACACGTCTG
EDN3	ACTCTGGACGTCAGCAGTAA	GCATGAGCTTTGGATGGTGG
EFNA1	CTGTACCTGGTGGAGCATGA	GGTGGATGGGTTTGGAGATGT
ELMO2	AGGCAGCCGTGTCTGTGTTT	CAAGGAGCTGGGCGTTAGCA
EPHB2	GTGTGTAACAGACGGGGGTT	CATGTGGCCACTGGTGTAGT
F2RL3	CATCCTCAGTGCCCAATGCT	GGGTTTTGGGGAGCAGAGTT

FOXF1	TCTCGCTCAACGAGTGCTTC	GTTCATCATGCTGTACATGGGC
GATA2	GACTCGCTGCTCAAGTCTGTC	CGCTTTTGTCCGCCTGGT
GATA3	CCTAAGGTGGTTGTGCTCGG	CACAGGCTGCAGGAATAGGG
GATA5	CGGGTCCGGTCATTCTGAAA	CATCGCATAGAAGTGGGGCT
GNA11	AAGAGGGTTGGTGCCAGAAG	CCTCAGAGATGCCACAAGCA
GNAQ	GATCTCAAAGCTTGAAGAAGCTGA	AACAGCGAAGCCACAAACCT
GPER1	GGCCGTCATTCCAGACAGCA	GTGTGTGCAGCTCCCGAGTC
GYS1	GACGAATGGGGCGACAACTA	CCGAAATACACCTTGCAGCC
HIF1A	GCAGTTCCGCAAGCCCTGAA	GGCAGTGGTAGTGGTGGCAT
HSPG2	ACACCTGTGAGGCCATGAAC	GGGCCTCGTTGTGGGAC
INSR	AAAACCTCTTCAGGCACTGGT	CGTCACATTCCCAACATCGC
ITGA3	GTGGCTTCACCCAGAACACT	GTGCAGGATGAAGCTGCCTA
ADM2	GTTATGGGTCAGCCTCTCCG	CTGAGATTCTGCACCTGGCA
ASIC2	GAGTGGAGCCGCCAGTTAC	GCCAGTGGCCGGCATAGTA
BAI1	ACTCCTTCCTCGAGTCCACG	GTACTCCACGGAGAAGTCGT
BNIP3	AGCAATAATGGGAACGGGGG	TCTTGGAGCTACTCCGTCCA
C2CD3	AACGAAAAGGCCAAGGGTCT	TGGACCACAACGAATAGCGT
C8A	CAGAGAGTAAGACGGGCAGC	GCAGATGGTTCCCCCAAACT
CACNA1B	GCGGGTCCTCTACAAGCAAT	TGATGCGCTTCGCGTATTTG
CACNA1C	CCCATGCCAACATGAATGCC	GACTGTGGAGATGGTCGCAT
CACNA1D	GGACCAACTTCTCAGCCGAA	TGCTCTTGGCGTATTGCTGA
CACNB3	TGTATGACGACTCCTACGTGC	CACTCCTCATCCAGTACGCC

CACNG4	CCATCGGCACCGACTACTG	CCGGAAGCAGTGCCCTTTAT
CACNG8	CATCGCCATCAGCACTGACTA	GACGCCTCTTTTCAACCCTTC
CAMK2A	GTGGCCCGGGAGTATTACAG	CTCCCCCTCCACCTCTATGG
CAPZA2	TCTGGAGGAGCAGTTGTCTG	CTTTCCATTTCCCAAGTCGCC
CD40	CTGTACGAGTGAGGCCTGTG	CAGGGCTCGCAGATGGTATC
CDX2	CATGTACCCTAGCTCCGTGC	GCGTAGCCATTCCAGTCCTC
ITIH1	AAGAGCAGCGAGAAGCGAC	CATAGTGGGCGAAGCGAGAG
JAM3	ATCCAGCAATCGAACCCCAG	TCCCCAGTATTTCTGCACGAC
KCNE2	CAATTGGCGCCAGAACACAA	GGTCATTGGAGTGTTCCCGT
KCNIP3	GCCGGCTAAGGAAGTGACAA	AGCTCACTGTCGCTGCTATC
KCNJ12	CATCGTGTCATCGGAGGAGG	GTCCACACAGGTGGTGAACA
KCNJ5	CGCGATTATGTCCCCATTGC	CAGGGTGGTGAAGAGGTCAC
KCNK15	CTGTGCACCCTGTGTTACCTG	GTGGCCGTACTCGATGGTAG
KCNMB4	AAGCCGAGGACAAGAGCATC	CAGGTGAAGGTGCACTCGAA
LEMD2	CGGGATGTCTACCGCAACAA	GGCCTCTTCCCGTAACCG
LGALS3BP	AACCCAAGGCGTGAACGATG	CTGGGTGGCGTTCTCGAAG
LUZP1	CTACAGAGTCTAAGCCGCCG	AGCACTTCAATCTCCGCCAA
MBD3	GCCACAGGGATGTCTTTTACT	CTGGCGGCTCTTGTTCATC
MYLK	TGGTCAGCCTGTTGTTTCCA	CTTGCAGGAGAATCGTCCCA
NCF1	GTACCCAGCCAGCACTATGT	GATCGCCCCTGCCTCAATAG
NCL	AAATGGCTCCTCCTCCAAAGG	GGGGAAACGACCACCTTCTT
NDRG4	GATTCCCTGAGGAGAAGCCG	TTTGTGGTTGAGGCCCACAT

NDUFS6	GCACACTGGCCAGGTTTATG	GCTATCACCCGAGTCTCCAC
NRARP	ACATGACCAACTGCGAGTTCA	CGTCGATGACCGACTGGTG
NTRK3	ATGTCTCTCTTTGCCCAGCC	CTGAATCCTGCCCTTCCAGG
PAX6	CAGAACAGTCACAGCGGAGT	GTCTGATGGAGCCAGTCTCG
PDLIM3	TTACACCAGGAAGCAAGGCG	TTGAGACACAGCTGGTGAGC
PDPK1	CCAGTCCAGCGTGGTGTTAT	AACTTGAAGTCCTCAGGCCG
PHF21A	TTGCAGACTCTACAGGAGGC	CAGATTGTGGCAATGGCTGT

All 112 candidate genes related to blood vessels and their qRT-PCR primers are exhibited above. Red highlights 9 VSMC related genes. GAPDH is used as endogenous control.

Supplemental Table 5 Sequences for siRNAs, miRNA mimics, miRNA agomir and qRT-PCR primers

Sequences for siRNAs		
p53	siRNA-1	GTACCACCATCCACTACAA
	siRNA-2	AGAGAATCTCCGCAAGAAA
	siRNA-3	GGAGTATTTGGATGACAGA
MYLK	siRNA-1	GACGGGAACTGCTCTTTAA
	siRNA-2	CTAAGACCATTCGCGATTT
	siRNA-3	GCAAGGCTGTCAACAGTCT
Sequences for miRNA mimics		
hsa-miR-1204 mimics	Sence 5'-UCGUGGCCUGGUCUCCAUUAU-3'<br>Antisence 5'-AAUGGAGACCAGGCCACGAUU-3'	
miRNA control	Sence 5'-UUCUCCGAACGUGUCACGUTT-3'<br>Antisence 5'-ACGUGACACGUUCGGAGAATT-3'	
Sequences for miRNA agomir		
micrON™ hsa-miR-1204 agomir	Sence 5'-UCGUGGCCUGGUCUCCAUUAU-3'<br>Antisence 5'-AUAAUGGAGACCAGGCCACGA-3'	
Primers sequences for qRT-PCR		
Primer	Forward	Reserve
IL-6	ACCCCCAGGAGAAGATTCCA	GCCTCTTTGCTGCTTTCACA
IL-8	AAGGTGCAGTTTTGCCAAGG	CCCAGTTTTCCTTGGGGTCC
MCP-1	CAGCCAGATGCAATCAATGCC	TGGAATCCTGAACCCACTTCT
CXCL-1	ACATCCAAAGTGTGAACGTGA	ATGGGGGATGCAGGATTGAG
CXCL-2	TGTGAAGGTGAAGTCCCCCG	CGATGCGGGGTTGAGACAA

IGFBP-3	CGCGCCAGGAAATGCTAGTG	TCAACTTTGTAGCGCTGGCT
TNF-α	CCTCTCTCTAATCAGCCCTCTG	GAGGACCTGGGAGTAGATGAG
MMP-9	GCAATGCTGATGGGAAACCC	TCGCTGGTACAGGTCGAGTA
p53	TCAGATAGCGATGGTCTGGC	CTCATAGGGCACCACCACAC
pri-mir-1204	GGCACAAGGGCCCAACT	TCCCTCTGGGAATTCAATGG
PVT1 response element	TGCATACTGGCAGCGACAAG	TTCGCTATGACCACAGGACTGT
p21 3' response element	CTGTCCTCCCCGAGGTCA	ACATCTCAGGCTGCTCAGAGTCT

Supplemental Table 6 The catalog of proprietary sequences

Name	Vendor or Source	Catalog#
Bulge-Loop^{TM} hsa-miR-98-3p Forward Primer	Guangzhou RiboBio Co.,Ltd	miR8000154
Bulge-Loop^{TM} hsa-miR-124-3p Forward Primer	Guangzhou RiboBio Co.,Ltd	miR8003823
Bulge-Loop^{TM} hsa-miR-143-3p Forward Primer	Guangzhou RiboBio Co.,Ltd	ssD2012112029
Bulge-Loop^{TM} hsa-miR-145-5p Forward Primer	Guangzhou RiboBio Co.,Ltd	ssD809230848
Bulge-Loop^{TM} hsa-miR-182-3p Forward Primer	Guangzhou RiboBio Co.,Ltd	ssD809231590
Bulge-Loop^{TM} hsa-miR-376b-5p Forward Primer	Guangzhou RiboBio Co.,Ltd	miR8007911
Bulge-Loop^{TM} hsa-miR-382-5p Forward Primer	Guangzhou RiboBio Co.,Ltd	ssD809231046
Bulge-Loop^{TM} hsa-miR-411-3p Forward Primer	Guangzhou RiboBio Co.,Ltd	ssD809231666
Bulge-Loop^{TM} hsa-miR-1204 Forward Primer	Guangzhou RiboBio Co.,Ltd	ssD809230725
Bulge-Loop^{TM} hsa-miR-1179 Forward Primer	Guangzhou RiboBio Co.,Ltd	ssD809230713
Bulge-Loop^{TM} mmu-miR-1204 Forward Primer	Guangzhou RiboBio Co.,Ltd	miR8007714
Bulge-Loop^{TM} miR-Reverse Primer	Guangzhou RiboBio Co.,Ltd	ssD089261711
Bulge-Loop^{TM} U6-Forward Primer	Guangzhou RiboBio Co.,Ltd	ssD0904071006
Bulge-Loop^{TM} U6-Reverse Primer	Guangzhou RiboBio Co.,Ltd	ssD0904071007
micrON^{TM} agomir Negative Control	Guangzhou RiboBio Co.,Ltd	miR4N0000001-4
hsa-miR-1204 miRCURY LNA miRNA Detection probe	QIAGEN	QIAGEN.339111-YD00611999-BGC
hsa-miR-1204 miRCURY LNA miRNA Inhibitor	QIAGEN	QIAGEN.339204-YI04100452

Supplemental Figures

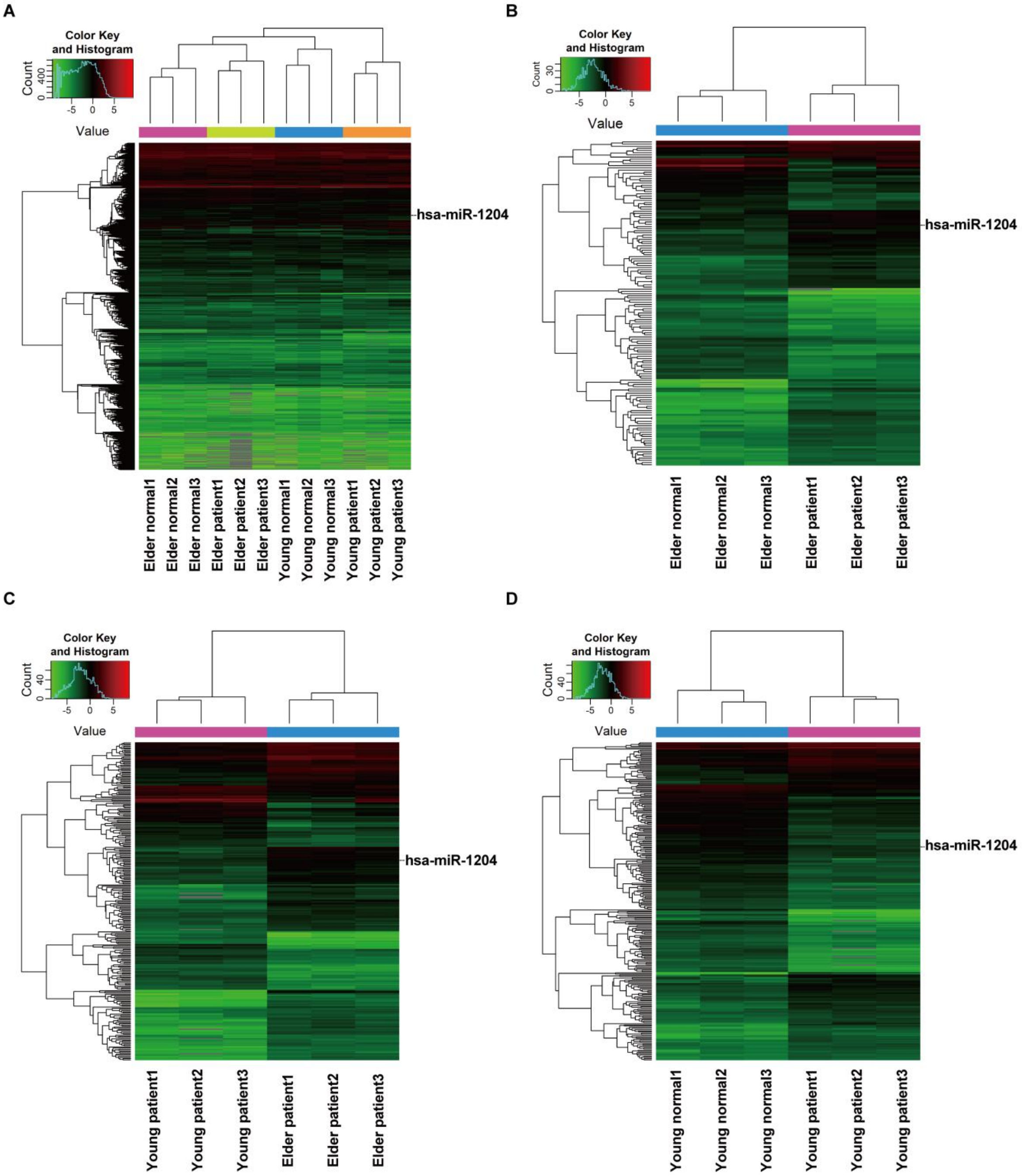

Supplemental Figure 1. miRNA expression profiles between healthy subjects and patients with aortic aneurysm and dissection (AAD) at different age stages using miRNA microarray

A. Heat map showing miRNA expression profiles in 4 groups: young normal (young healthy

subjects), elder normal (elder healthy subjects), young patients and elder patients, by performing microarray analysis on RNA extracts from plasma. B-D. Heat map showing specific elevation of miR-1204 in elder patient groups.

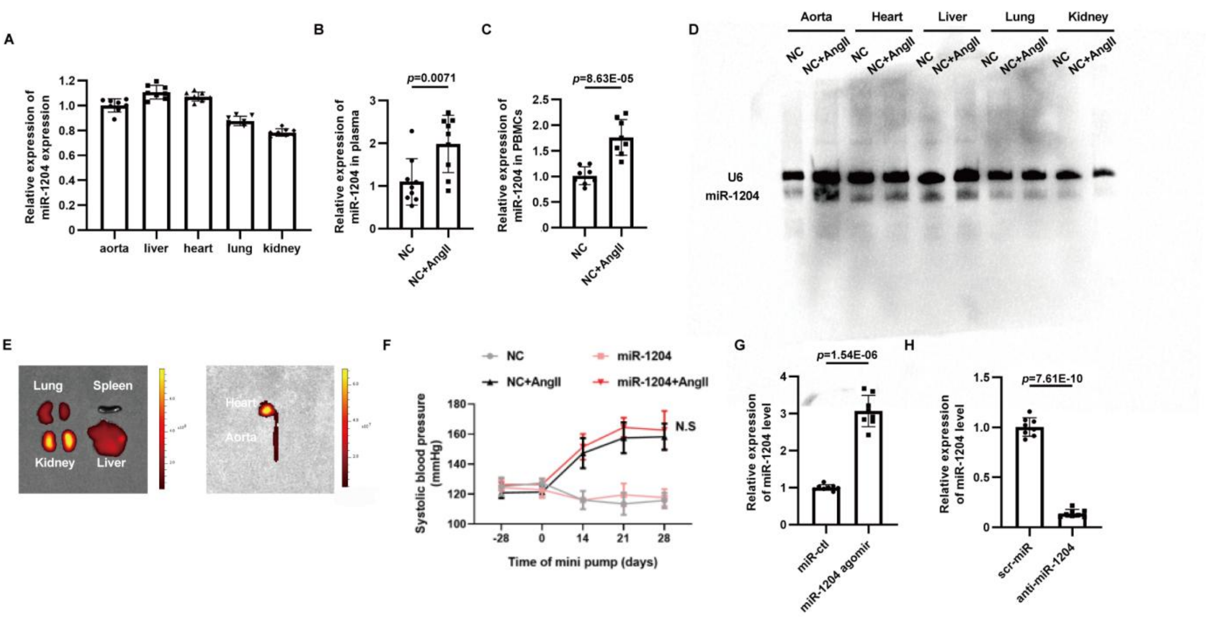

Supplemental Figure 2. miR-1204 aggravates angiotensin II (AngII)-induced aortic aneurysm and dissection (AAD) formation

A. The baseline expression level of mmu-miR-1204 in major organs was determined by qRT-PCR in C57BL/6 mice. n=8 biological replicates. B. mmu-miR-1204 expression in the plasma was determined by qRT-PCR in AngII-induced AAD mice or control mice. n=9 biological replicates. Statistical analysis was performed by two-tailed Student's t-test. C. mmu-miR-1204 expression in the peripheral blood mononuclear cells (PBMCs) was determined by qRT-PCR in AngII-induced AAD mice or control mice. n=8 biological replicates. Statistical analysis was performed by two-tailed Student's t-test. D. Northern blotting was employed to assess the expression of mmu-miR-1204 in major organs of AngII-induced AAD mice or control mice. E. Fluorescent images of C57BL/6 mice obtained at 6 h after tail vein injection with Cy5-labeled miR-1204 agomir revealed that miR-1204 agomir accumulated in the aorta. F. Systolic blood pressure measured using a tail-cuff method. n=17 biological replicates for miR-1204+AngII group. n=19 biological replicates for other

groups. Statistical analysis was performed by two-tailed repeated-measures ANOVA with Bonferroni's multiple comparisons test. G. Overexpression of miR-1204 *in vivo* was confirmed by qRT-PCR. n=8 biological replicates. Statistical analysis was performed by two-tailed Student's t-test with Welch's correction. H. Inhibition of miR-1204 *in vivo* was confirmed by qRT-PCR. n=8 biological replicates. Statistical analysis was performed by two-tailed Student's t-test with Welch's correction. Data were presented as mean ± SD. N.S indicates not significant. Source data are provided as a Source Data file.

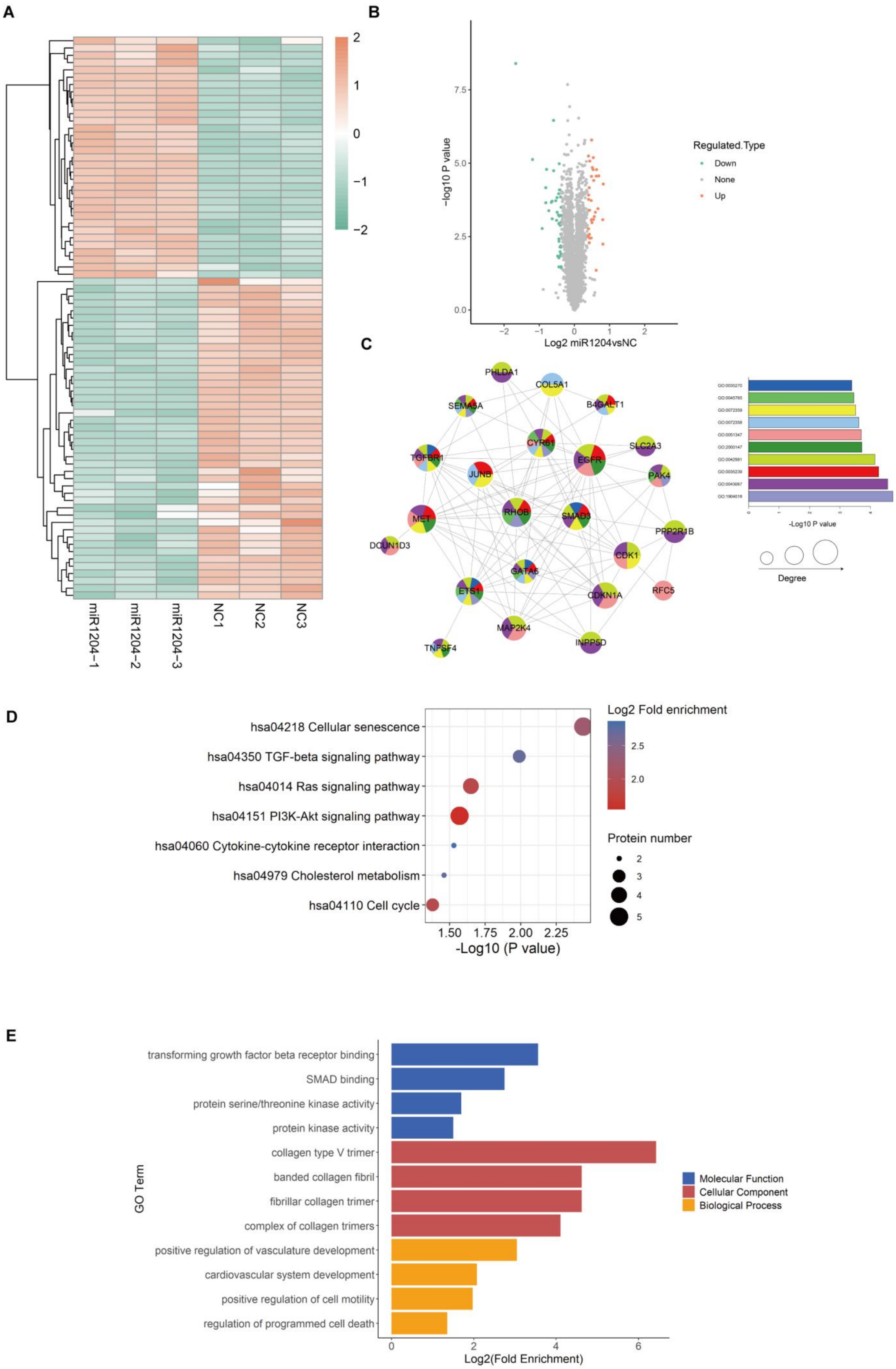
A
miR1204−1
miR1204−2
miR1204−3
NC1
NC2
NC3
2
1
0
−1
−2
B
Regulated.Type
Down
None
Up
−log10 P value
Log2 miR1204vsNC
C
PHLDA1
COL5A1
SEMA5A
B4GALT1
CYR61
TGFBR1
EGFR
SLC2A3
JUNB
PAK4
MET
RHOB
SMAD3
PPP2R1B
DCUN1D3
CDK1
GATA6
ETS1
CDKN1A
RFC5
MAP2K4
TNFSF4
INPP5D
-Log10 P value
Degree
D
hsa04218 Cellular senescence
hsa04350 TGF-beta signaling pathway
hsa04014 Ras signaling pathway
hsa04151 PI3K-Akt signaling pathway
hsa04060 Cytokine-cytokine receptor interaction
hsa04979 Cholesterol metabolism
hsa04110 Cell cycle
-Log10 (P value)
Log2 Fold enrichment
Protein number
E
transforming growth factor beta receptor binding
SMAD binding
protein serine/threonine kinase activity
protein kinase activity
collagen type V trimer
banded collagen fibril
fibrillar collagen trimer
complex of collagen trimers
positive regulation of vasculature development
cardiovascular system development
positive regulation of cell motility
regulation of programmed cell death
GO Term
Log2(Fold Enrichment)
Molecular Function
Cellular Component
Biological Process

Supplemental Figure 3. The protein profiles in vascular smooth muscle cells (VSMCs) were different among control and miR-1204 groups using tandem mass tag (TMT) based quantitative proteomics

A. Heat map of 77 proteins differentially expressed in overexpressing miR-1204 and control VSMCs. Adjusted $P<0.05$, fold change >1.3. B. Volcano plot, with 44 downregulated proteins (green) and 33 upregulated proteins (red) in overexpressing miR-1204 and control VSMCs. Adjusted $P<0.05$, fold change>1.3. Statistical analysis was performed using two-tailed Student's t-test. C. Network analysis of proteins involved in biological process, visualized with STRING. D. KEGG analysis of differentially expressed proteins in overexpressing miR-1204 VSMCs. Statistical analysis was performed by a hypergeometric test. E. GO analysis of differentially expressed proteins in overexpressing miR-1204 VSMCs. Statistical analysis was performed by a hypergeometric test.

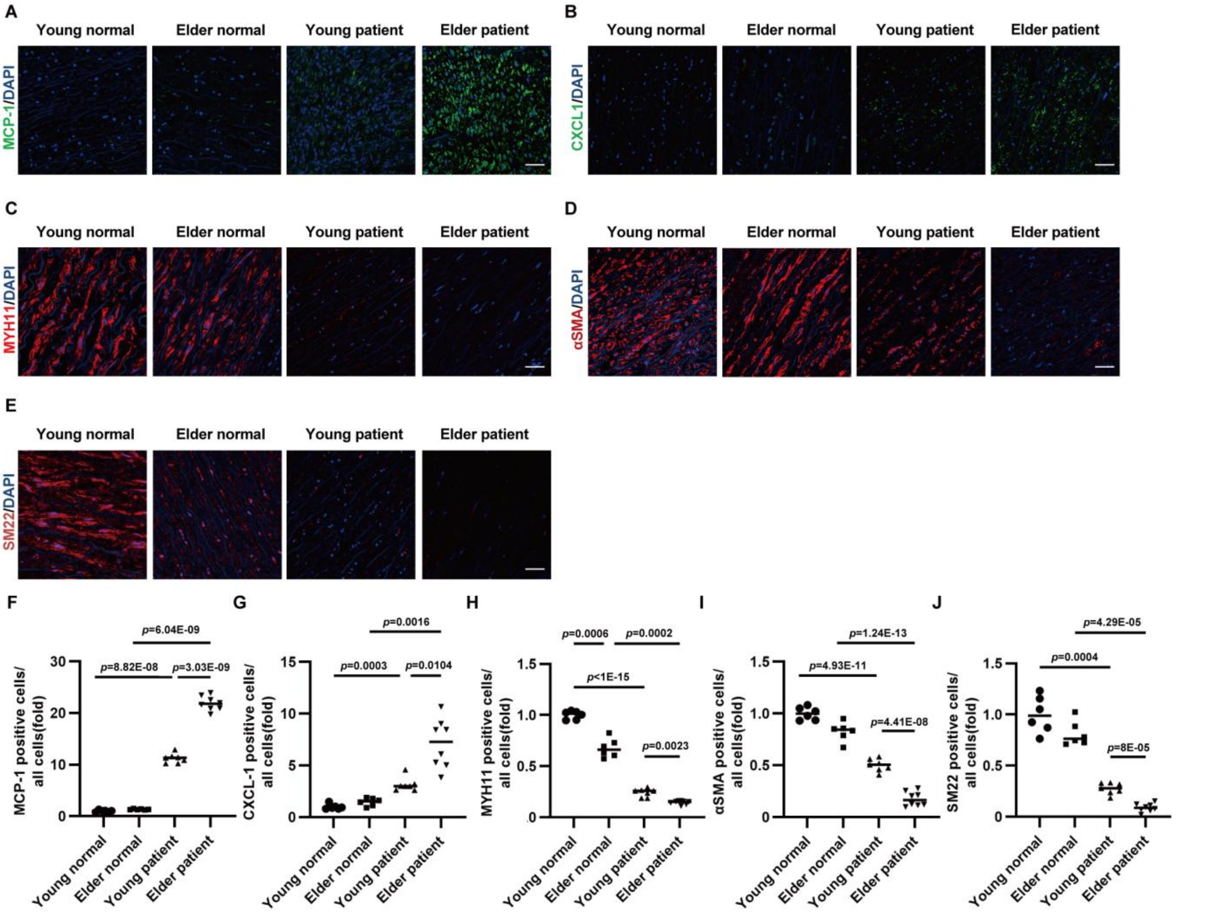

Supplemental Figure 4. Senescence-associated secretory phenotype (SASP) components accumulation and contractile phenotype loss were detected in human aortic aneurysm and dissection (AAD) tissues in an age-related manner

A–E. Representative images of immunofluorescence staining of SASP components, monocyte chemotactic protein 1 (MCP-1), CXC chemokine ligands 1 (CXCL1), and contractile markers, myosin heavy chain 11 (MYH11), α-smooth muscle actin (α-SMA), and smooth muscle protein 22 (SM22) in aortas of healthy subjects and patients with aortic aneurysm and dissection. Scale bar represents 20 μm. F–J. Quantification of immunofluorescence staining of SASP components, MCP-1, CXCL1, and contractile markers, MYH11, α-SMA, and SM22 in aortas of healthy subjects and patients with aortic aneurysm

and dissection. n=6 (young normal), n=6 (elder normal), n=7 (young patient), n=8 (elder patient). Statistical analysis was performed by two-tailed Welch's ANOVA followed by Dunn's multiple comparisons for F, G, H, J, by one-way ANOVA followed by Tukey's multiple comparisons for I. Data were presented as mean ± SD. Source data are provided as a Source Data file.

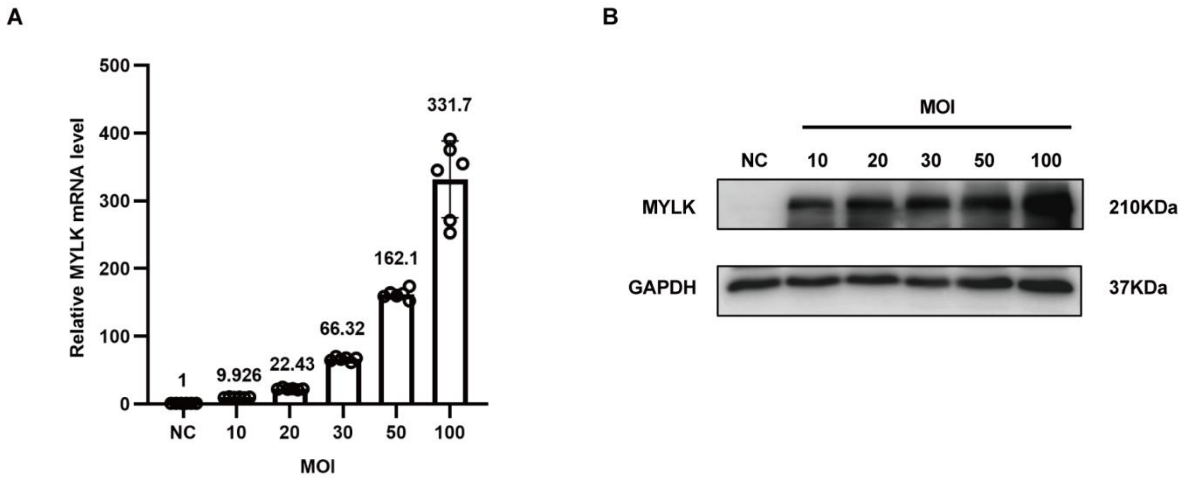

Supplemental Figure 5. Verification of myosin light chain kinase (MYLK) overexpression

A. The mRNA levels of MYLK in vascular smooth muscle cells (VSMCs) transfected with negative control (NC) adenovirus or MYLK adenovirus at various multiplicities of infection (MOI). n=biological replicates. B. Immunoblotting for the expression of MYLK in VSMCs transfected with NC adenovirus or MYLK adenovirus at various MOI. Source data are provided as a Source Data file.

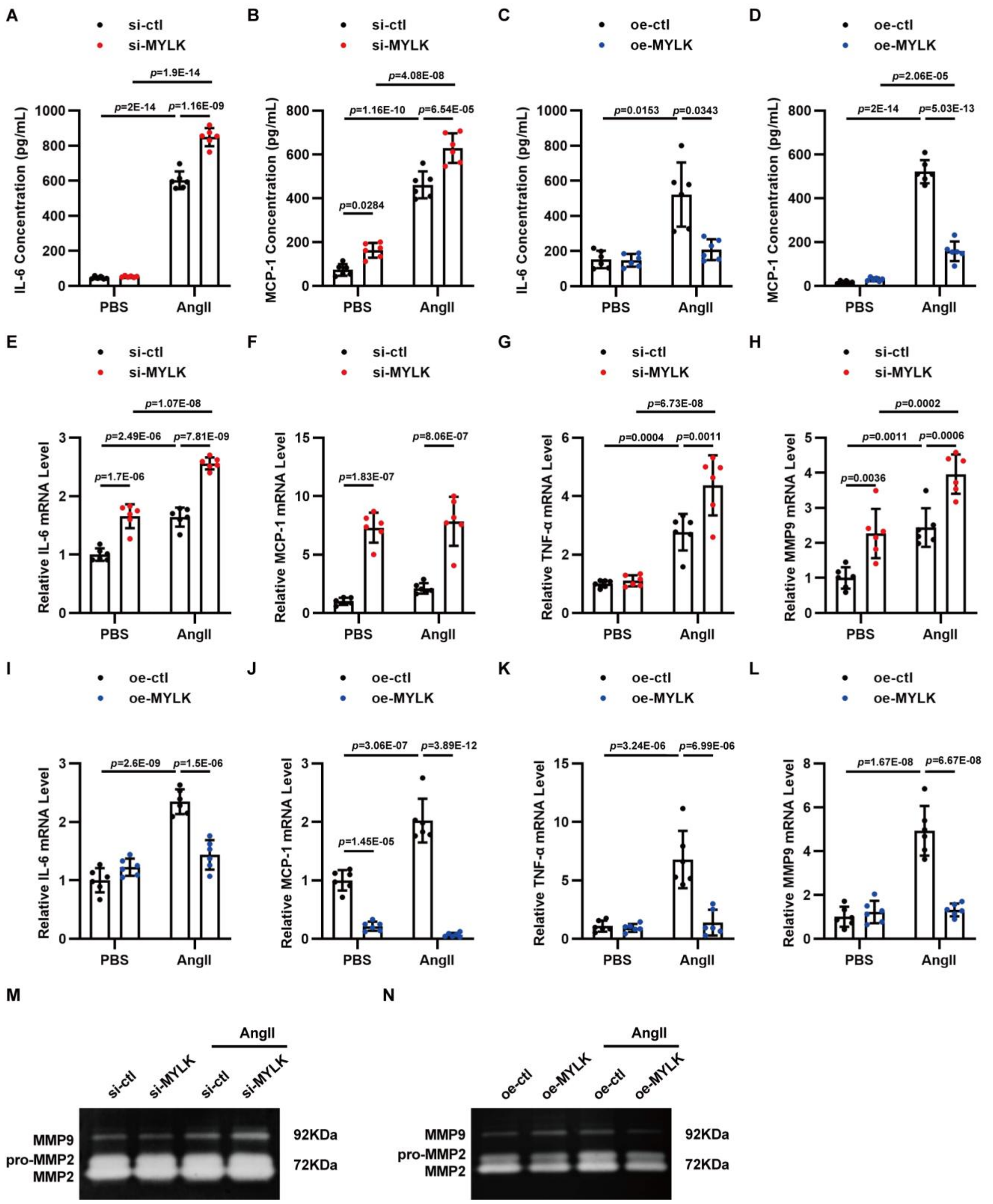

Supplemental Figure 6. Myosin light chain kinase (MYLK) modulates vascular smooth muscle cell (VSMC) senescence-associated secretory phenotype (SASP)

A–B. Interleukin 6 (IL-6) and monocyte chemotactic protein 1 (MCP-1) concentration in the culture supernatant of VSMCs after silencing of MYLK combined with or without angiotensin II (AngII) treatment. n=6 biological replicates. Statistical analysis was performed

by one-way ANOVA followed by Tukey's multiple comparisons. C–D. IL-6 and MCP-1 concentration in the culture supernatant of VSMCs after overexpression of MYLK combined with or without AngII treatment. n=6 biological replicates. Statistical analysis was performed by two-tailed Welch's ANOVA followed by Dunn's multiple comparisons for C, by one-way ANOVA followed by Tukey's multiple comparisons for D. E–H. Quantification of mRNA levels of SASP components, including IL-6, MCP-1, tumor necrosis factorα (TNF-α), and matrix metalloproteinase 9 (MMP9) in VSMCs after silencing of MYLK combined with or without AngII treatment. n=6 biological replicates. Statistical analysis was performed by one-way ANOVA followed by Tukey's multiple comparisons. I–L. Quantification of mRNA levels of SASP components, including IL-6, MCP-1, TNF-α, and MMP9 in VSMCs after overexpression of MYLK combined with or without AngII treatment. n=6 biological replicates. Statistical analysis was performed by one-way ANOVA followed by Tukey's multiple comparisons. M–N. Representative images of gelatin zymography in the culture supernatant of VSMCs after silencing or overexpression of MYLK combined with or without AngII treatment. The experiment was repeated 3 times independently with similar results. Data were presented as mean ± SD. Source data are provided as a Source Data file.

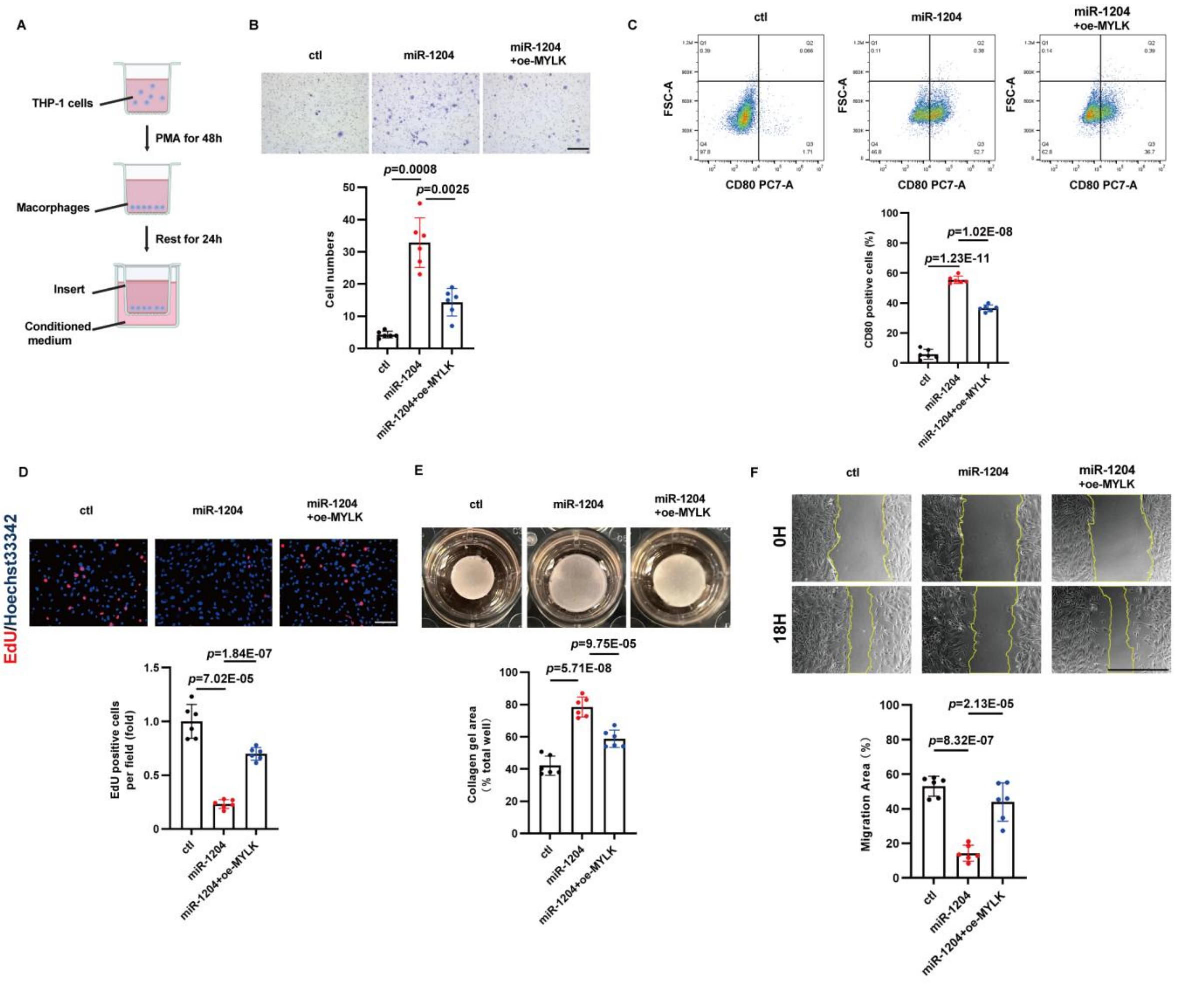

Supplemental Figure 7. Myosin light chain kinase (MYLK) ameliorates the effect of miR-1204 on macrophages and vascular smooth muscle cell (VSMC) characteristics

A. Scheme of transwell system, which comprised a 24-well culture insert in the upper part plating macrophages and a 24-well plate in the lower part adding conditioned medium from miR-1204 overexpressing VSMCs. PMA was used to induce THP1 cell differentiation. B. Representative images and quantitative analysis of the migration of macrophages in transwell chambers. n=6 biological replicates. Statistical analysis was performed by two-tailed Welch's ANOVA followed by Dunn's multiple comparisons. The scale bar represents 100 μm. C. Representative flow cytometry charts and quantification of CD80 positive macrophages in indicated groups. n=6 biological replicates. Statistical analysis was performed by one-way

ANOVA followed by Tukey's multiple comparisons. D. Representative images and quantitative analysis of 5-ethynyl-2'-deoxyuridine (EdU) proliferation assay. n=6 biological replicates. Statistical analysis was performed by two-tailed Welch's ANOVA followed by Dunn's multiple comparisons. The scale bar represents 100 μm. E. Representative images and quantitative analysis of the collagen gel contraction assay. n=6 biological replicates. Statistical analysis was performed by one-way ANOVA followed by Tukey's multiple comparisons. F. Representative images and quantitative analysis of the wound-healing assay. n=6 biological replicates. Statistical analysis was performed by one-way ANOVA followed by Tukey's multiple comparisons. The scale bar represents 1000 μm. Data were presented as mean ± SD. Source data are provided as a Source Data file.

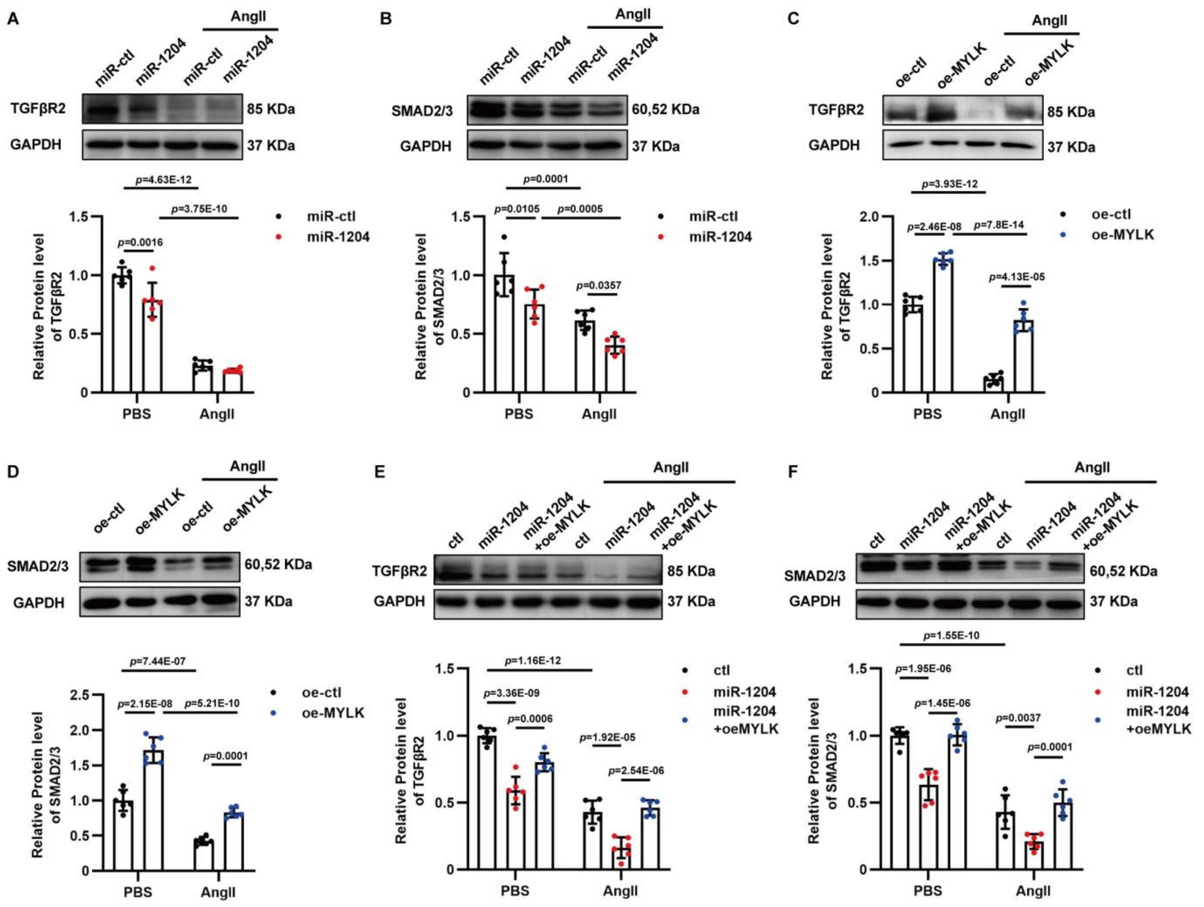

Supplemental Figure 8. MYLK ameliorates miR-1204-induced damage via transforming growth factor-β (TGF-β) signaling pathway in vascular smooth muscle cells (VSMCs)

A–B. Representative plots (upper panel) and quantification (below panel) of immunoblot analysis of transforming growth factor-β receptor 2 (TGFβR2) and drosophilamothers against decapentaplegic protein2/3 (SMAD2/3) in VSMCs transfected with miR-1204 mimics or miR-control (miR-ctl) combined with or without angiotensin II (AngII) treatment. n=6 biological replicates. Statistical analysis was performed by one-way ANOVA followed by Tukey’s multiple comparisons. C–D. Representative plots (upper panel) and quantification (below panel) of immunoblot analysis of TGFβR2 and SMAD2/3 in VSMCs after silencing of MYLK combined with or without AngII treatment. n=6 biological replicates. Statistical

analysis was performed by one-way ANOVA followed by Tukey's multiple comparisons. E–F. Overexpression of MYLK restored miR-1204-mediated TGFβR2 and SMAD2/3 decrease with or without AngII treatment in VSMCs. n=6 biological replicates. Statistical analysis was performed by one-way ANOVA followed by Tukey's multiple comparisons. Data were presented as mean ± SD. Source data are provided as a Source Data file.

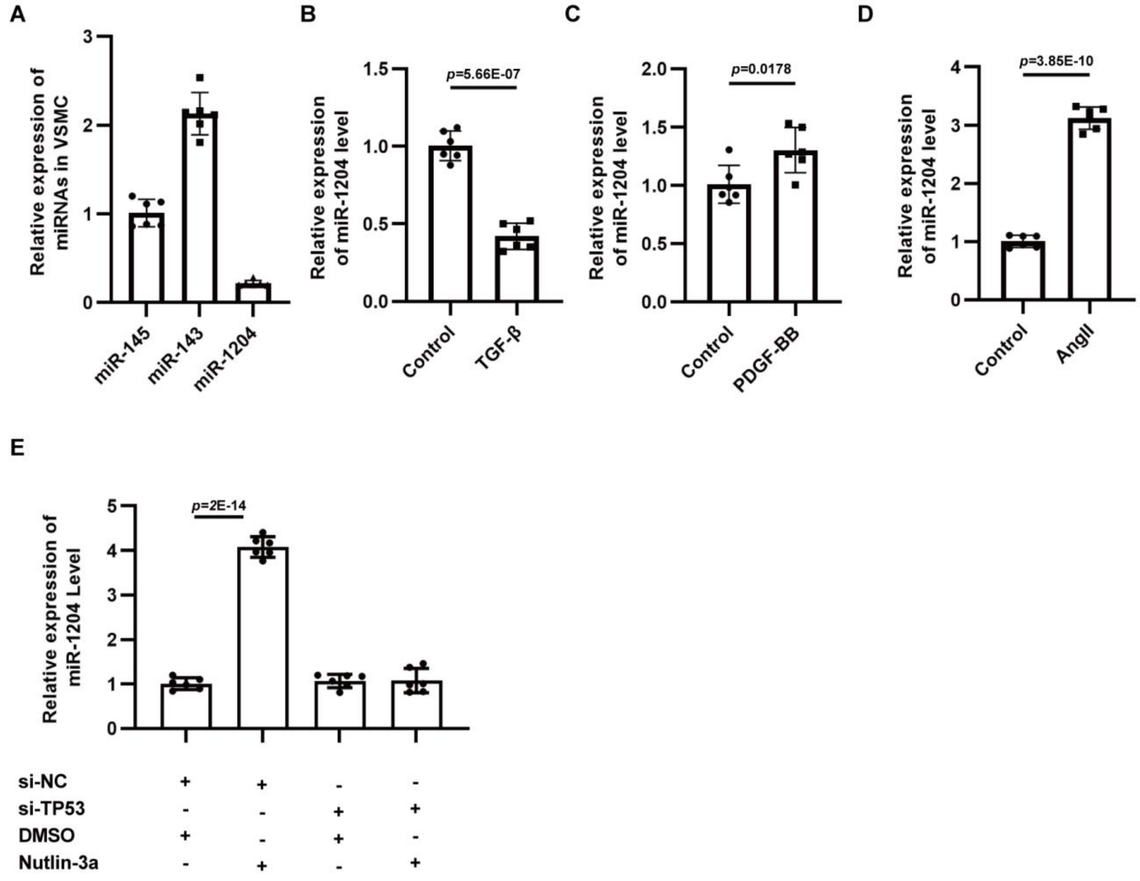

Supplemental Figure 9. Quantification of mature miR-1204 levels in vascular smooth muscle cells (VSMCs) in response to various stimuli

A. Quantification of mature miR-1204 levels in VSMCs compared with miR143/145. n=6 biological replicates. B. Quantification of mature miR-1204 levels in VSMCs treated with transforming growth factor-β1 (TGF-β1, 5ng/ml). n=6 biological replicates. Statistical analysis was performed by unpaired two-tailed Student's t-test. C. Quantification of mature miR-1204 levels in VSMCs treated with platelet-derived growth factor-BB (PDGF-BB, 10ng/ml). n=6 biological replicates. Statistical analysis was performed by unpaired two-tailed Student's t-test. D. Quantification of mature miR-1204 levels in VSMCs treated with angiotensin II (AngII, 1×10^{-6}M). n=6 biological replicates. Statistical analysis was performed by unpaired two-tailed Student's t-test. E. Quantification of mature miR-1204

levels in VSMCs treated with nutlin-3a with or without silencing TP53. n=6 biological replicates. Statistical analysis was performed by one-way ANOVA followed by Tukey's multiple comparisons. Data were presented as mean ± SD. Source data are provided as a Source Data file.
