# Supplementary material for: Aging aggravates aortic aneurysm and dissection via miR-1204-MYLK signaling axis in mice
Source: Nat Commun. 2024 Jul 16;15:5985. doi: 10.1038/s41467-024-50036-2 (PMC11252124; doi:10.1038/s41467-024-50036-2)
Supplement: Supplementary file 3 — Reporting Summary [file 41467_2024_50036_MOESM3_ESM.pdf]

nature portfolio

Corresponding author(s): Jingsong Ou

Last updated by author(s): Apr 8, 2024

Reporting Summary

Nature Portfolio wishes to improve the reproducibility of the work that we publish. This form provides structure for consistency and transparency in reporting. For further information on Nature Portfolio policies, see our Editorial Policies and the Editorial Policy Checklist.

Statistics

For all statistical analyses, confirm that the following items are present in the figure legend, table legend, main text, or Methods section.

n/a	Confirmed	
☐	☒	The exact sample size (*n*) for each experimental group/condition, given as a discrete number and unit of measurement
☐	☒	A statement on whether measurements were taken from distinct samples or whether the same sample was measured repeatedly
☐	☒	The statistical test(s) used AND whether they are one- or two-sided<br>*Only common tests should be described solely by name; describe more complex techniques in the Methods section.*
☒	☐	A description of all covariates tested
☐	☒	A description of any assumptions or corrections, such as tests of normality and adjustment for multiple comparisons
☐	☒	A full description of the statistical parameters including central tendency (e.g. means) or other basic estimates (e.g. regression coefficient) AND variation (e.g. standard deviation) or associated estimates of uncertainty (e.g. confidence intervals)
☐	☒	For null hypothesis testing, the test statistic (e.g. *F*, *t*, *r*) with confidence intervals, effect sizes, degrees of freedom and *P* value noted<br>*Give P values as exact values whenever suitable.*
☒	☐	For Bayesian analysis, information on the choice of priors and Markov chain Monte Carlo settings
☒	☐	For hierarchical and complex designs, identification of the appropriate level for tests and full reporting of outcomes
☒	☐	Estimates of effect sizes (e.g. Cohen's *d*, Pearson's *r*), indicating how they were calculated

Our web collection on statistics for biologists contains articles on many of the points above.

Software and code

Policy information about availability of computer code

Data collection	The following standard software provided by instrument suppliers was used for data collection:<br>WB: Amersham Imager 600 (GE Healthcare).<br>qRT-PCR: Bio-rad CFX96 system.<br>Flow Cytometry: Beckman Coulter CytoFlex with CytExpert software<br>Immunofluorescence: laser scanning confocal microscope (Zeiss LSM 780, Germany)<br>Histology: KFBIO scanner (KFBIO, KF-PRO-020)<br>Ultrasound: Vevo 2100 ultrasound system (Visual Sonics, Toronto, Canada).<br>Microarray: Axon GenePix 4000B microarray scanner (Axon Instruments, CA, USA).<br>ChIP-seq: Illumina NovaSeq 6000 (Novogene, Beijing, China).<br>Tandem mass tagging (TMT) proteomics: Q ExactiveTM HF-X (Thermo, USA).<br>Mice fluorescence imaging: Xenogen IVIS Spectrum (Caliper Life Sciences, MA, USA).
Data analysis	To plot the data and for statistical anaysis was used GraphPad Prism version 9.0 and IBM SPSS Statistics (version 25, IBM Corp., Armonk, NY, USA).<br>To quantification of histology images and IF composites was used ImageJ 2.1.0.<br>For Flow Cytometry data analysis was used FlowJo 10.8.1.<br>For quantification of WB bands densities was used Adobe Photoshop 2020 software.<br>To analyze qRT-PCR data was used Bio-rad CFX Manager.<br>For analyze ultrasound data was used Vevo LAB Software Package.

For analyze microarray data was used GenePix Pro 6.0 software.
For analyze proteomics data was used eggNOG v5.0.0, KEGG mapper v5.0, STRING v11.0.
For analyze ChIP-seq data was used Bowtie2 (Version 2.5.0) MACS2 (version 2.1.0) ChIPseeker(version 3.17)

For manuscripts utilizing custom algorithms or software that are central to the research but not yet described in published literature, software must be made available to editors and reviewers. We strongly encourage code deposition in a community repository (e.g. GitHub). See the Nature Portfolio guidelines for submitting code & software for further information.

Data

Policy information about availability of data

All manuscripts must include a data availability statement. This statement should provide the following information, where applicable:

- Accession codes, unique identifiers, or web links for publicly available datasets
- A description of any restrictions on data availability
- For clinical datasets or third party data, please ensure that the statement adheres to our policy

The data that support the findings of this study are available within the article and its supplementary information/Source data or from the corresponding authors on reasonable request. The mass spectrometry proteomics data have been deposited to the ProteomeXchange Consortium via the PRIDE partner repository with the dataset identifier PXD048658 (https://proteomecentral.proteomexchange.org/cgi/GetDataset?ID=PXD048658). The microarray data have been deposited to the GEO with the dataset identifier GSE253747 (https://www.ncbi.nlm.nih.gov/geo/query/acc.cgi?acc=GSE253747). The ChIP-seq data have been deposited to the GEO with the dataset identifier GSE255157 (https://www.ncbi.nlm.nih.gov/geo/query/acc.cgi?acc=GSE255157). Source data are provided with this paper.

Research involving human participants, their data, or biological material

Policy information about studies with human participants or human data. See also policy information about sex, gender (identity/presentation), and sexual orientation and race, ethnicity and racism.

Reporting on sex and gender	The sex characterstics for human participants are detailed in Table 1 and supplementary Table 1.
Reporting on race, ethnicity, or other socially relevant groupings	The population characteristics of human participants are shown in Table 1 and Supplemental Table 1.
Population characteristics	The subjects were divided into four groups, namely young normal (< 50 years old), elder normal (> 50 years old), young patient (< 50 years old) and elder patient (> 50 years old) based on age and disease states. Other characteristics that were recorded include smoking, hypertension, diabetes mellitus, connective tissue diseases, triglyceride, total cholesterol, low-density lipoprotein, high-density lipoprotein and uric acid. The demographic data and basic clinical parameters are shown in Table 1 and Supplemental Table 1.
Recruitment	A total of 272 patients (116 young patients and 156 elder patients) diagnosed with aortic aneurysm and dissection (AAD) and 158 healthy subjects (70 young healthy subjects, 88 elder healthy subjects) were recruited for plasma miRNA detection. Patients were diagnosed as AAD based on computed tomography angiography. Patients who suffered from infectious disease, tumor, renal failure, or had undergone surgery or severe trauma within the preceding 3 months were excluded. Healthy subjects were enrolled who undergoing physical examination during the same period. There are no known biases affecting patient recruitment, selection of samples, or in analysis.
Ethics oversight	This study was approved by the Ethics Review Board of the First Affiliated Hospital, Sun Yat-sen University, and the Second Xiangya Hospital of Central South University. Informed consent was obtained from all the subjects enrolled into this study.

Note that full information on the approval of the study protocol must also be provided in the manuscript.

Field-specific reporting

Please select the one below that is the best fit for your research. If you are not sure, read the appropriate sections before making your selection.

[x] Life sciences   [ ] Behavioural & social sciences   [ ] Ecological, evolutionary & environmental sciences

For a reference copy of the document with all sections, see nature.com/documents/nr-reporting-summary-flat.pdf

Life sciences study design

All studies must disclose on these points even when the disclosure is negative.

Sample size	The sample size in each experiment is detailed in the figure legend and represented as data point in each graph. To achieve a 90% power for detecting a difference between groups with a significance level of 0.05, PASS software 15 (NCSS LLC., Kaysville, U.T., USA) was used to estimate the animal sample size based on the AAD incidence in the pre-experiment. For in vitro experiments, sample sizes were determined based on our previous experience. At least three independent biological replicates were performed.
Data exclusions	No data were excluded
Replication	The experiments were carried out at least in 3 independent experiment and the results were constent between experiments.

Randomization	For in vivo experiments, mice were randomly allocated into experimental groups. For cell line-based experiments, randomization was not required because all samples were analyzed equally.
Blinding	In our ultrasound analysis, all measurements were gathered by one observer to limit bias, while another independent observer analyzed the records. In the rest of the experiments, the investigators were not blinded in regard to allocation of samples during experiments and outcome assessment. However, the outcomes were quantitative and not subjective.

Reporting for specific materials, systems and methods

We require information from authors about some types of materials, experimental systems and methods used in many studies. Here, indicate whether each material, system or method listed is relevant to your study. If you are not sure if a list item applies to your research, read the appropriate section before selecting a response.

Materials & experimental systems

n/a	Involved in the study	
☐	☒	Antibodies
☐	☒	Eukaryotic cell lines
☒	☐	Palaeontology and archaeology
☐	☒	Animals and other organisms
☒	☐	Clinical data
☒	☐	Dual use research of concern
☒	☐	Plants

Methods

n/a	Involved in the study	
☐	☒	ChIP-seq
☐	☒	Flow cytometry
☒	☐	MRI-based neuroimaging

Antibodies

Antibodies used

MYH11 (1/100, Cat.ab53219, UK, Abcam),
α-SMA (1/200, Cat.19245, USA, Cell Signaling Technology),
SM22 (1/100, Cat.ab14106, UK, Abcam),
MYLK (1/50, Cat.sc-365352, USA, Santa Cruz Biotechnology),
MCP-1 (1/50, Cat.ab214819, UK, Abcam),
IL-6 (1/50, Cat.ab233706, UK, Abcam),
CXCL1 (1/100, Cat.12335-1-AP, USA, Proteintech)
CD68 (1/100, Cat.ab283667, UK, Abcam)

Anti-mouse IgG H&L (Alexa Fluor® 488)(1/1000, Cat.4408, USA, Cell Signaling Technology)
Anti-rabbit IgG H&L (Alexa Fluor® 555)(1/1000, Cat.4413, USA, Cell Signaling Technology)

TGFβR2 (1/1000, Cat.79424, USA, Cell Signaling Technology)
SMAD2/3 (1/1000, Cat.8685, USA, Cell Signaling Technology)
p53 (1/1000, Cat.2524, USA, Cell Signaling Technology)
p21 (1/1000, Cat.2947, USA, Cell Signaling Technology)
p16 (1/1000, Cat.92803, USA, Cell Signaling Technology)
p-γH2Ax (1/1000, Cat.5438, USA, Cell Signaling Technology)
p-MDM2 (1/1000, Cat.ab131355, UK, Abcam)
MDM2 (1/1000, Cat.ab226939, UK, Abcam)
GAPDH (1/1000, Cat.60004-1-Ig, USA, Proteintech)

Anti-mouse IgG HRP-linked antibody (1/10000, Cat.7076, USA, Cell Signaling Technology)
Anti-rabbit IgG HRP-linked antibody (1/10000, Cat.7074, USA, Cell Signaling Technology)

anti-p53 antibody (Cat.ab1101, UK, Abcam)
IgG (Cat.2729, USA, Cell Signaling Technology)

PE/Cyanine7 anti-human CD80 antibody (Cat.305218, USA, Biolegend)

Validation

The antibodies used in the study were validated by the corresponding manufacturer and/or were widely used in the literature. Commercial antibodies were employed in accordance with the manufacturers ' recommendations as provided on their datasheets and offcial websites.

MYH11 (1/100, Cat.ab53219, UK, Abcam), https://www.abcam.cn/products/primary-antibodies/smooth-muscle-myosin-heavy-chain-11-antibody-ab53219.html, (129 citations)
α-SMA (1/200, Cat.19245, USA, Cell Signaling Technology), https://www.cellsignal.cn/products/primary-antibodies/a-smooth-muscle-actin-d4k9n-xp-174-rabbit-mab/19245, (527 citations)
SM22 (1/100, Cat.ab14106, UK, Abcam), https://www.abcam.cn/products/primary-antibodies/taglntransgelin-antibody-ab14106.html, (383 citations)

MYLK (1/50, Cat.sc-365352, USA, Santa Cruz Biotechnology), https://www.scbt.com/p/mylk-antibody-a-8?requestFrom=search, (12 citations)
MCP-1 (1/50, Cat.ab214819, UK, Abcam), https://www.abcam.cn/products/primary-antibodies/mcp1-antibody-epr21025-ab214819.html, (6 citations)
IL-6 (1/50, Cat.ab233706, UK, Abcam), https://www.abcam.cn/products/primary-antibodies/il-6-antibody-epr21711-ab233706.html, (41 citations)
CXCL1 (1/100, Cat.12335-1-AP, USA, Proteintech), https://www.ptgcn.com/products/CXCL1-Antibody-12335-1-AP.htm, (45 citations)
CD68 (1/100, Cat.ab283667, UK, Abcam), https://www.abcam.cn/products/primary-antibodies/cd68-antibody-epr23917-164-bsa-and-azide-free-ab283667.html

Anti-mouse IgG H&L (Alexa Fluor® 488)(1/1000, Cat.4408, USA, Cell Signaling Technology), https://www.cellsignal.cn/products/secondary-antibodies/anti-mouse-igg-h-l-f-ab-2-fragment-alexa-fluor-488-conjugate/4408, (553 citations)
Anti-rabbit IgG H&L (Alexa Fluor® 555)(1/1000, Cat.4413, USA, Cell Signaling Technology), https://www.cellsignal.cn/products/secondary-antibodies/anti-rabbit-igg-h-l-f-ab-2-fragment-alexa-fluor-555-conjugate/4413, (386 citations)

TGFβR2 (1/1000, Cat.79424, USA, Cell Signaling Technology), https://www.cellsignal.cn/product/productDetail.jsp?productId=79424, (20 citations)
SMAD2/3 (1/1000, Cat.8685, USA, Cell Signaling Technology), https://www.cellsignal.cn/products/primary-antibodies/smad2-3-d7g7-xp-rabbit-mab/8685, (549 citations)
p53 (1/1000, Cat.2524, USA, Cell Signaling Technology), https://www.cellsignal.cn/products/primary-antibodies/p53-1c12-mouse-mab/2524, (1358 citations)
p21 (1/1000, Cat.2947, USA, Cell Signaling Technology), https://www.cellsignal.cn/products/primary-antibodies/p21-waf1-cip1-12d1-rabbit-mab/2947, (1982 citations)
p16 (1/1000, Cat.92803, USA, Cell Signaling Technology), https://www.cellsignal.cn/products/primary-antibodies/p16-ink4a-d3w8g-rabbit-mab/92803, (26 citations)
p-γH2Ax (1/1000, Cat.5438, USA, Cell Signaling Technology), https://www.cellsignal.cn/products/primary-antibodies/phospho-histone-h2a-x-ser139-tyr142-antibody/5438, (38 citations)
p-MDM2 (1/1000, Cat.ab131355, UK, Abcam), https://www.abcam.cn/products/primary-antibodies/mdm2-phospho-s166-antibody-ab131355.html, (5 citations)
MDM2 (1/1000, Cat.ab226939, UK, Abcam), https://www.abcam.cn/products/primary-antibodies/mdm2-antibody-chip-grade-ab226939.html, (4 citations)
GAPDH (1/1000, Cat.60004-1-Ig, USA, Proteintech), https://www.ptgcn.com/products/GAPDH-Antibody-60004-1-Ig.htm, (9284 citations)

Anti-mouse IgG HRP-linked antibody (1/10000, Cat.7076, USA, Cell Signaling Technology), https://www.cellsignal.cn/products/secondary-antibodies/anti-mouse-igg-hrp-linked-antibody/7076, (8738 citations)
Anti-rabbit IgG HRP-linked antibody (1/10000, Cat.7074, USA, Cell Signaling Technology), https://www.cellsignal.cn/products/secondary-antibodies/anti-rabbit-igg-hrp-linked-antibody/7074, (14786 citations)

anti-p53 antibody (Cat.ab1101, UK, Abcam), https://www.abcam.cn/products/primary-antibodies/p53-antibody-do-1-chip-grade-ab1101.html, (151 citations)
IgG (Cat.2729, USA, Cell Signaling Technology), https://www.cellsignal.cn/products/primary-antibodies/normal-rabbit-igg/2729, (2141 citations)

PE/Cyanine7 anti-human CD80 antibody (Cat.305218, USA, Biolegend), https://www.biolegend.com/en-gb/products/pe-cyanine7-anti-human-cd80-antibody-6174, (17 citations)

Eukaryotic cell lines

Policy information about cell lines and Sex and Gender in Research

Cell line source(s)	Primary human aortic VSMCs were purchased from ScienCell (HASMC, Cat. No. 6110, Carlsbad, California, USA). HEK-293T cells were purchased from Procell (Cat.CL-0005, China)
Authentication	HASMC have been authenticated by immunofluorescence with antibodies to specific to α-SMA by ScienCell. HEK-293T have been authenticated by STR profiling by Procell.
Mycoplasma contamination	Cell lines were mycoplasma negative.
Commonly misidentified lines (See ICLAC register)	No commonly misidentified lines were used in the study.

Animals and other research organisms

Policy information about studies involving animals; ARRIVE guidelines recommended for reporting animal research, and Sex and Gender in Research

Laboratory animals	Male and female C57BL/6 mice were used for the experiments. 4-month-old C57BL/6J mice were used for Angiotensin II-induced AAD model. Three-week-old C57BL/6J mice were used for BAPN-induced AAD model. Mice were housed in cages with 50% humidity, 20℃ temperature, with a 12 h light/dark cycle, kept on standard rodent chow diet and water. Special attention was paid to animal welfare and to minimize their suffering.

Wild animals | No wild animals were used in the study.

Reporting on sex | Our research findings apply to both male and female. In the original submission, we used male mice for this study. After we received the editor and reviewers' comments, we performed additional experiments with both male and female mice.

Field-collected samples | No field-collected samples were used in the study.

Ethics oversight | All animal experiments were approved by the Ethics Review Board and Animal Research Committee of the First Affiliated Hospital, Sun Yat-sen University.

Note that full information on the approval of the study protocol must also be provided in the manuscript.

Plants

Seed stocks | *Report on the source of all seed stocks or other plant material used. If applicable, state the seed stock centre and catalogue number. If plant specimens were collected from the field, describe the collection location, date and sampling procedures.*

Novel plant genotypes | *Describe the methods by which all novel plant genotypes were produced. This includes those generated by transgenic approaches, gene editing, chemical/radiation-based mutagenesis and hybridization. For transgenic lines, describe the transformation method, the number of independent lines analyzed and the generation upon which experiments were performed. For gene-edited lines, describe the editor used, the endogenous sequence targeted for editing, the targeting guide RNA sequence (if applicable) and how the editor was applied.*

Authentication | *Describe any authentication procedures for each seed stock used or novel genotype generated. Describe any experiments used to assess the effect of a mutation and, where applicable, how potential secondary effects (e.g. second site T-DNA insertions, mosiacism, off-target gene editing) were examined.*

ChIP-seq

Data deposition

☒ Confirm that both raw and final processed data have been deposited in a public database such as GEO.

☒ Confirm that you have deposited or provided access to graph files (e.g. BED files) for the called peaks.

Data access links
May remain private before publication. | The ChIP-seq data have been deposited to the GEO with the dataset identifier GSE255157 (https://www.ncbi.nlm.nih.gov/geo/query/acc.cgi?acc=GSE255157).

Files in database submission | miR-ctl
miR-ctl-input
miR-1204
miR-1204-input

Genome browser session
(e.g. UCSC) | https://www.igv.org/ (IGV_Win_2.4.14)

Methodology

Replicates | one biological replicates

Sequencing depth |

Sample	total_reads	uniquely_maped_reads	length	paried
miR-ctl-input	44352942	43424075	150	PE
miR-ctl	54702146	22587803	150	PE
miR-1204-input	48049736	47059188	150	PE
miR-1204	52983132	47263425	150	PE

Antibodies | anti-p53 antibody (Cat.ab1101, UK, Abcam)

Peak calling parameters | MACS2 (-p 1e-2 --nomodel --shift 0 --extsize 150 --keep-dup all -B --SPMR)

Data quality | FastQC v0.11.9

Software | Bowtie2 (Version 2.5.0) MACS2 (version 2.1.0) ChIPseeker(version 3.17)

Flow Cytometry

Plots

Confirm that:

- [x] The axis labels state the marker and fluorochrome used (e.g. CD4-FITC).
- [x] The axis scales are clearly visible. Include numbers along axes only for bottom left plot of group (a 'group' is an analysis of identical markers).
- [x] All plots are contour plots with outliers or pseudocolor plots.
- [x] A numerical value for number of cells or percentage (with statistics) is provided.

Methodology

Sample preparation	After different treatments, macrophages were washed and stained with anti-CD80 PE/Cyanine7 antibody (Cat.305218, USA, Biolegend) for 45 min at 4℃. After the staining, cells were washed and analyzed. More detailed are provided in methods section.
Instrument	Beckman Coulter CytoFlex with CytExpert software
Software	FlowJo 10.8.1
Cell population abundance	The percentage and abundance of each population is detailed in the main text and in Figure S7.
Gating strategy	Unstained cells were used to define negative cell populations and set gates for analysis.

- [ ] Tick this box to confirm that a figure exemplifying the gating strategy is provided in the Supplementary Information.
